# Systemic intermittent parathyroid hormone treatment improves osseointegration of press-fit inserted implants in cancellous bone: A canine study

**DOI:** 10.3109/17453674.2012.702388

**Published:** 2012-08-25

**Authors:** Henrik Daugaard, Brian Elmengaard, Troels Torp Andreassen, Anders Lamberg, Joan Elisabeth Bechtold, Kjeld Soballe

**Affiliations:** ^1^Orthopaedic Research Laboratory, Department of Orthopedic Surgery, Aarhus University Hospital, Aarhus, Denmark; ^2^Institute of Anatomy, Faculty of Health Sciences, Aarhus University, Aarhus, Denmark; ^3^Orthopaedic Biomechanics Laboratory, Excelen Center for Bone and Joint Research and Minneapolis Medical Research Foundation, Minneapolis, MN, USA

## Abstract

**Background and purpose:**

Intermittent administration of parathyroid hormone (PTH) has an anabolic effect on bone, as confirmed in human osteoporosis studies, distraction osteogenesis, and fracture healing. PTH in rat models leads to improved fixation of implants in low-density bone or screw insertion transcortically.

**Material and methods:**

We examined the effect of human PTH (1–34) on the cancellous osseointegration of unloaded implants inserted press-fit in intact bone of higher animal species. 20 dogs were randomized to treatment with human PTH (1–34), 5 μg/kg/day subcutaneously, or placebo for 4 weeks starting on the day after insertion of a cylindrical porous coated plasma-sprayed titanium alloy implant in the proximal metaphyseal cancellous bone of tibia. Osseointegration was evaluated by histomorphometry and fixation by push-out test to failure.

**Results:**

Surface fraction of woven bone at the implant interface was statistically significantly higher in the PTH group by 1.4 fold with (median (interquartile range) 15% (13–18)) in the PTH group and 11% (7–13) in control. The fraction of lamellar bone was unaltered. No significant difference in bone or fibrous tissue was observed in the circumferential regions of 0–500, 500–1,000, and 1,000–2,000 μm around the implant. Mechanically, the implants treated with PTH showed no significant differences in total energy absorption, maximum shear stiffness, or maximum shear strength.

**Interpretation:**

Intermittent treatment with PTH (1–34) improved xhistological osseointegration of a prosthesis inserted press-fit at surgery in cancellous bone, with no additional improvement of the initial mechanical fixation at this time point.

Long-term survival of total joint replacements depends on secure and lasting fixation. In uncemented implants, this is achieved by firm anchorage of the implant at surgery, by subsequent osseointegration, and from optimal bone stock at the peri-implant trabecular bone (Bauer and Schils 1999). Press-fitting of the prosthetic component into the prepared cancellous bone bed is done to maximize bone-implant contact and to stabilize the prosthesis at surgery. However, due to implant porosity and the trabecular nature of bone structure, only a small part of the prosthetic surface may initially be in direct contact with bone. Within the first weeks of implantation, bone resorption and remodeling occurs in peri-implant bone with micromotion of the implant (Ryd et al. 1995, Hilding et al. 2000). Subsequent ingrowth into the porous coating may vary considerably, ranging from 10% to 48% (Cook et al. 1988, Engh et al. 1993).

Considering the importance of initial implant fixation for long-term survival, it would be desirable to accelerate formation of new bone within the implant porosity by incorporation of cancellous bone. The bone-forming agent parathyroid hormone (PTH) has provided an exciting new option for potential improvement of osseointegration. PTH administered intermittently as the native polypeptide (1–84) or as the N-terminal-fragment (1–34) induces bone formation by increasing the mass and strength of both cancellous (Ejersted et al. 1995, Jerome et al. 1995, Zhang et al. 1998, Oxlund et al. 2002) and cortical bone (Oxlund et al. 1993, Ejersted et al. 1994) with variable impact on the various bone envelopes (Ejersted et al. 1994, Nishida et al. 1994, Jerome et al. 1995). The effect has been confirmed in human fracture-prevention studies (Neer et al. 2001, Orwoll et al. 2003). Experimental distraction osteogenesis (Seebach et al. 2004, Aleksyniene et al. 2006, 2009) and fracture healing show increased callus volume and mechanical strength (Kim et al. 1996, Andreassen et al. 1999, Alkhiary et al. 2005, Manabe et al. 2007, Barnes et al. 2008, Nozaka et al. 2008, Aspenberg et al. 2009). Until now, research on fixation of prostheses by adjuvant PTH administration has been confined to experimental rodent settings with pathological low bone density (Shirota et al. 2003, Gabet et al. 2006) or involving transcortical screw insertion (Skripitz et al. 2000b, Skripitz and Aspenberg 2001b).

It is of interest to determine the effect of PTH on the cancellous osseointegration of implants. This has not been studied previously. In the present study, we used a well-established experimental dog model for joint replacement with cylindrical implants inserted press-fit in intact cancellous metaphyseal bone to represent the cancellous portion of a press-fit joint replacement implant. We tested the hypothesis that systemic human PTH (1–34) mainly improves the early mechanical implant fixation and bone ongrowth at the bone-implant interface, and secondary peri-implant bone volume.

## Material and methods

### Test animal

The Institutional Animal Care and Use Committee of the Minneapolis Medical Research Foundation approved the study based on a pilot protocol. The animals were operated and observed at the Animal Care facilities of the Research Foundation at the Hennepin County Medical Center, Minnesota, according to the regulations of the National Institutes of Health. Processing of the bone specimens, mechanical testing, and histomorphometry were done at the Orthopedic Research Laboratory of Aarhus University Hospital, Denmark. An experimental randomized dog study was done using 20 fully grown, skeletally mature American Hound dogs (HRP Covance Research Products, Kalamazoo, MI), with a mean age (range) of 13.8 (11.6–20.0) months and mean weight of 24.8 (21.0–28.9) kg. All animals were male and were purpose-bred for research. The animals were fed a nutritionally complete commercial standard diet for dogs in research. The animals were housed individually but socialized in groups with 1–2 hours of daily exercise within the large housing room. The daily routines in the stall followed natural circadian rhythm. Other unrelated studies on PTH treatment were conducted in this set of test animals, at different bone sites and with a different implant model. The studies involved anatomically separate sites with different implant settings and are considered independent.

### Hormone administration

The 20 test animals were randomly divided in 2 groups of 10 by a staff member other than the surgeon. The animals were randomly allocated to the treatment group postoperatively. The intervention group was injected subcutaneously with human PTH (PTH (1–34), research grade; Bachem Holding, Bubendorf, Switzerland), 5 µg/kg body weight daily (with the exception of 1 day), between 8 a.m. and 10 a.m. Injections were started on the day after surgery and continued until the end of the 4-week observation period. On day 11, PTH (1–34) was changed from research grade to GMP-grade (Bachem, Torrance, CA) with unaltered dosage due to concern about trifluoroacetic acid (TFA) content. The control group was injected with drug vehicle in similar volume. The animals were weighed once a week, and injections were adjusted to body weight in 0.5-kg increments. The drug vehicle for the PTH was prepared in a sterile environment using (as vehicle) heat-inactivated (56°C, 1 h) 2% dog serum (S-1757; Sigma-Aldrich) in 0.9% NaCl adjusted to pH 5 (Andreassen et al. 1999). This vehicle was passed through a 0.22-µm Micropore sterilization filter. The drugs for the whole study were prepared all at once and stored at –20°C until use.

### Implants

Biomet Inc. (Warsaw, IN) manufactured the implants for unrestricted use. Test implants involved a press-fit implant device. Implants were custom-made and cylindrical, with a porous coated surface of plasma-sprayed titanium alloy (Ti6Al4V ELI, ASTM F136) superimposed on an implant core of similar titanium composition. The plasma-spray coating was similar to that used for clinical applications of the manufacturer’s prostheses. Implant diameter was mean 6.13 (SD 0.14) mm and length was 10 mm. Before autoclave sterilization, the implants were cleaned in an ultrasonic bath of trichlorethylene with final baths of ethanol. Besides implants used for implantation, 4 additional implants were randomly chosen and evaluated with quantitative topography (The Danish Technological Institute, Copenhagen, Denmark) (Roughness profilometer, Somicronic Surfascan, 3CS, Hommel Somicronic, Saint-Andr-De-Corcy, France). The implant roughness—expressed as mean (range)—was as follows. Ra (arithmetic average of the deviation from the mean line over a sampling length) = 55.6 (51.1–63.5) μm, Rz (average difference in height between the 5 highest peaks and the 5 lowest valleys) = 267 (238–299) μm, Rq (root mean square value of the profile departure ) = 67 (61–76) μm, and Rmax (maximum peak-to-valley height) = 267 (238–299) μm.

### Implant site

A randomly selected implant was inserted press-fit into the extraarticular metaphyseal cancellous bone site of the left proximal tibia perpendicular to the anterior medial plane. A radiograph was obtained postoperatively to verify correct implant position and to screen for skeletal abnormality. All implants were handled by the same surgeon and showed primary stability after insertion. Postoperatively, the dogs were allowed unrestricted weight bearing.

### Surgery

Surgery was performed under sterile conditions and under general anesthesia. Premedication included atrophine (0.4 mg/mL, 1 mL/4.5 kg subcutaneously) and acepromazine (10 mg/mL, 0.1 mL/4.5 kg subcutaneously. Anesthesia was provided by 5% thiopental (8 mL intravenously) before intubation and maintenance by inhalation of 1.5% isoflurane. Postoperative pain management consisted of wound infiltration with bupivacain (2.5 mg/mL, 2 mL) and intramuscular injections of bupronex (buprenorphine; 0.3 mg/mL 0.01 mg/kg/daily for 3 days). The antibiotic rocephone (ceftriaxone) was administered prophylactically before surgery (1 g intravenously) and at 1 g/day intravenously for the first 3 days.

The anteromedial surface of the proximal tibia was exposed 4 cm distal to the tibial plateau. The cranial tibia muscle and medial collateral ligament were identified and the periosteum elevated in the implant insertion area. A 2.5-mm guide wire was inserted into the metaphyseal bone of the proximal tibia 12 mm from the femorotibial joint line, perpendicular to the anterior medial bone surface. Over the guide wire, a cannulated 5.9-mm drill was used to drill the cylindrical bone bed cavity. Immediately after drilling, the implant was inserted with light blows. At this site and with this method, the implant was completely surrounded by cancellous bone. The overlying soft tissue was closed in layers.

### Specimen preparation

**Figure 1.**
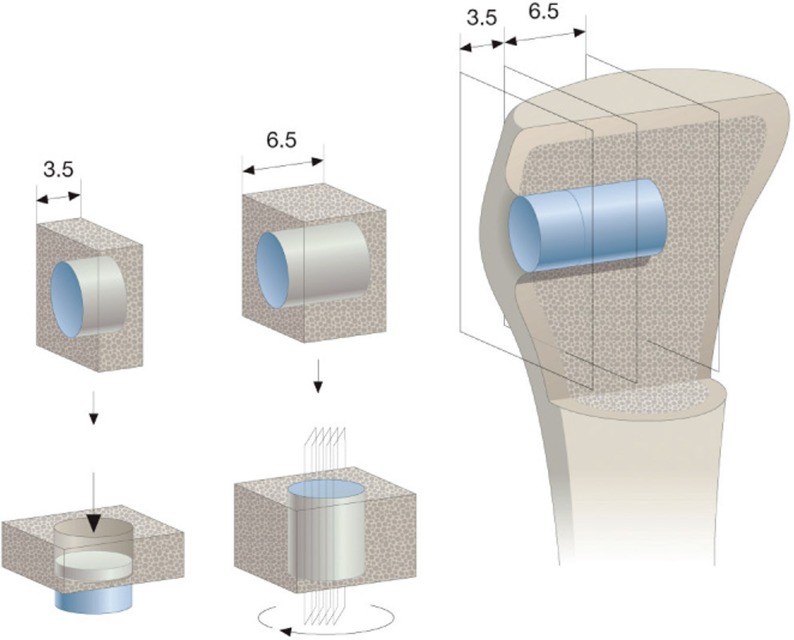
Sectioning technique. Implant in situ in the tibia metaphyseal bone is illustrated to the right. Inner part of 6.5 mm was used for histomorphometry using the vertical-section method applying 4 sections around implant center after random rotation around implant axis (center panel). Outer part of 3.5 mm was used for mechanical testing (left panel).

Joint fluid was obtained at euthanasia and subjected to culture to rule out infection. Bones with implants in situ were removed and frozen at –20°C until sectioning. All specimen preparation was done with blinding as to treatment group. 2 transverse bone-implant sections were cut perpendicular to the implant axis (Figure 1), using a water-cooled diamond-band saw and implant-based alignment post (Exact Apperatebau, Nordstedt, Germany). The first superficial 3.5-mm section for mechanical testing was stored at –20°C until testing. The inner 6.5-mm implant section was processed for un-decalcified histomorphometric evaluation with implant in situ. Specimens were dehydrated in graded ethanol (70–100%) containing 0.4% basic fuchsin followed by embedding in methylmethacrylate. The vertical uniform random (VUR) section technique was used with random rotation of the specimen blocks, and serial section around the center part and parallel to the implant axis using a hard-tissue microtome (KDG-95; MeProTech, Heerhugoward, the Netherlands) (Figure 1). 4 serial specimens were obtained from the center of the implant, parallel to implant axis, and spaced 400 µm apart—as the minimal space achieved due to kerf of the saw (Gotfredsen et al. 1989). The mean (SD) section thickness was 32 (7.1) µm. After specimen sectioning, the surface was counterstained with 2% light green for 2 min and rinsed in tap water. The depth of penetration of the stain was determined to be in the 4–10 µm range, as evaluated in the peri-implant bone that was not subjected to implantation. Counterstaining of the surface bone made it possible to establish a single-focus plane for sampling of tissue parameters, which reduces overestimation of tissue from the deeper parts of the section. The staining distinguishes bone at the surface of the specimen (green) as woven and lamellar bone, and fibrous tissue/marrow-like tissue (red). The specimen preparation and stereological sampling technique provides reliable results with minimal bias (Baddeley et al. 1986, Overgaard et al. 2000, Baas 2008).

### Histological analysis

**Figure 2.**
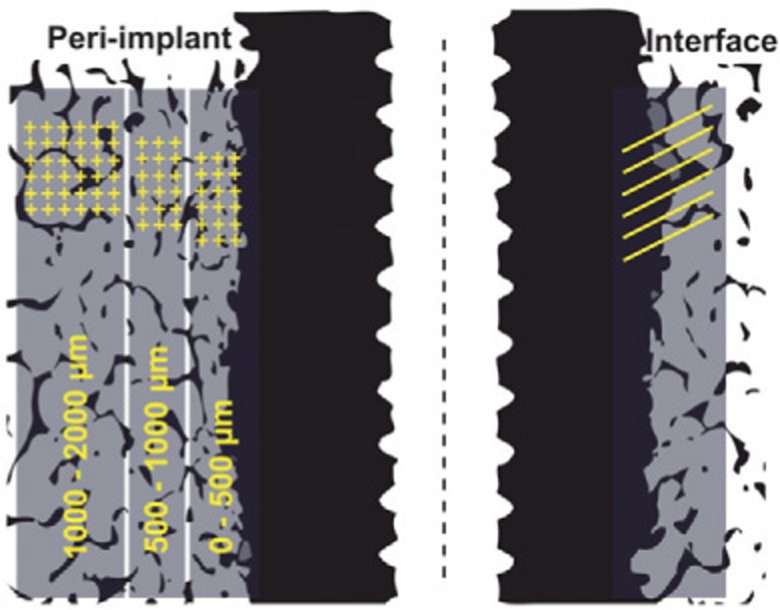
Histomorphometry – ROI. Region of interest (ROI) was defined on both sides of the implant, but on this schematic drawing illustrated on only one side. Tissue ongrowth (surface fraction) with interface tissue line counting (illustrated on right side) and peri-implant regions 0–500 µm, 500–1,000 µm, and 1,000–2,000 µm (volume fraction) with tissue point counting (illustrated on left side).

We quantified ongrowth of tissue at the implant surface and tissue volume fractions of tissue around the implant by static morphological histomorphometry (Figure 2). Computer-assisted histomorphometry was performed blind in random order of specimens with a light microscope (Olympus BX50) and stereological image analysis system (Cast version 2.1.4; Olympus). The computer software ensured stereological sampling by randomly displaying histological images and by a point-line overlay on the image. Region-of-interest (ROIs) was established at an objective/total magnification ×1.25/×34 and data were sampled at ×10/×300. Tissue ongrowth was defined as tissue directly at the implant surface and determined using the linear intercept technique with random disposed sine weighted lines. Peri-implant tissue volume fraction was determined using point counting with random point disposition. Concentric peri-implant regions defined host bone not subjected to implantation. These regions were determined as 0–500 µm, 500–1,000 µm, and 1,000–2,000 µm regions-of-interest from the implant. The implant surface was defined as the mean line of the rough implant topography along the implant axis. Ongrowth and volume parameters were sampled from 500 µm below the cleared (unsectioned) end of the implant along the whole length of the implant by meander sampling. The test system was calibrated to have at least 100 line interceptions or points counted for each parameter, as recommended previously (Gundersen and Jensen 1987, Overgaard et al. 2000). Each zone was evaluated independently during a specimen sampling session, and by the same person (HD). Bone discrimination between woven and lamellar bone was based on morphological characteristics. Woven bone showed anarchic distribution of cells and extracellular matrix with large ostecytes randomly oriented. Lamellar bone showed a homogeneous arrangement of cells with osteocytes being smaller, in lower numbers and distributed on more parallel straight lines. In case of uncertain bone morphology, polarized light was added and revealed the parallel lamellar structure of collagen in lamellar bone. The histomorphometric reproducibility was estimated from double measurements done by the same person (HD) using identical equipment and set-up (intra-observer variation). The coefficient of variance (CV) of tissues was determined at the interface and in the peri-implant regions. For bone the CV at the interface was 13%, in region 0–500 µm 9%, in region 500–1,000 µm, 8%, and in region 1,000–2,000 µm 9%. At the interface and in the same peri-implant regions CV for marrow tissue for all regions were 2% and for fibrous tissue in all regions 0%.

### Mechanical testing

**Figure 3.**
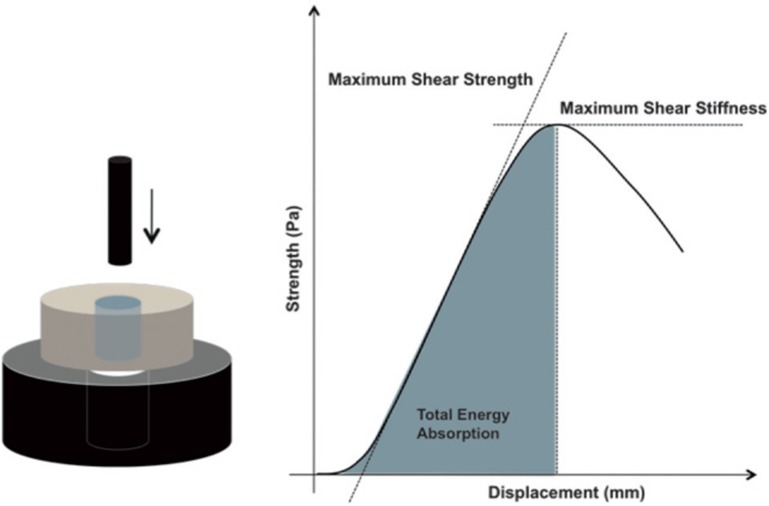
Mechanical testing. Left panel: Axial push-out test with specimen placed on metal platform with central opening. Specimen thickness = 3.5 mm, implant diameter = 6 mm, support hole diameter = 7.4 mm, preload = 2 N, and displacement velocity = 5 mm/min. Right panel: Load-displacement curve enabling calculation of ultimate shear strength (MPa), apparent shear stiffness (MPa/mm), and total energy absorption (J/m^2^).

The bone-implant interface was tested to failure by axial push-out test (MTS 858 Mini Bionix; MTS System Corporation, Minneapolis, MN; with MTS Test Star 790.00 software version 4.00). Testing was done blinded and all in one session. The specimens were placed on a metal support jig and the implant centered over a 7.4-mm circular opening, ensuring a distance of 0.7 mm between the implant and the support jig as recommended by Dhert (Dhert et al. 1992). Implants were pushed from the external side of the bone inwards. A preload of 2 N defined the standardized contact position for starting the test. At a displacement velocity of 5 mm/min, continuous load-displacement data were recorded and the 3 mechanical parameters achieved (Figure 3). Ultimate shear strength (MPa) was determined from the maximum force applied until failure of the bone-implant interface. Maximum shear stiffness (MPa/mm) was obtained from the slope of the linear section of the load-displacement curve, and total energy absorption (J/m^2^) was calculated as the area under the load-displacement curve until failure. All push-out parameters were normalized by the cylindrical surface area of the transverse implant section tested (π × diameter × length). Due to the destructive nature of the mechanical test, determination of reproducibility was not possible.

### Statistics

STATA statistical software was used (Stata 10.1; StataCorp, College Station, TX). The number of animals included was based on a sample size estimation for the unpaired study groups (Johansen 2002). The assumptions for sample size estimations were based on data from previous studies using similar models at our institution. With the minimal relevant difference of 80% in mechanical and histomorphometrical data, SD 50%, α = 0.05, and a power of 0.8, sample size was calculated to be 16, and 20 animals were included in the study.

Data were not assumed to be normally distributed and statistical analysis used the non-parametric two-sample Wilcoxon rank-sum (Mann-Whitney) test with assessment of the difference between treatment groups. Estimates are given as medians and interquartile ranges, and p < 0.05 was considered statistically signiﬁcant.

## Results

### Surgery

All animals were ambulatory on the day of surgery and fully weight bearing after 1 day of recovery. 2 animals in the PTH group were lost postoperatively (on days 6 and 8). Post mortem morbidity reports revealed ventricular hypertrophy and myositis in both. All other animals completed the 4-week observation period with no postoperative complications or abnormal serum calcium levels. No clinical infections were observed and no bacterial growth was detected in joint fluid cultures at euthanasia.

By the end of the observation period, 1 implant in the control group was excluded from histomorphometric and mechanical testing due to incorrect implant size at the time of surgery. 1 mechanical implant specimen in the control group showed incomplete implant exposure after sectioning and was excluded before testing.

### Histology

**Figure 4.**
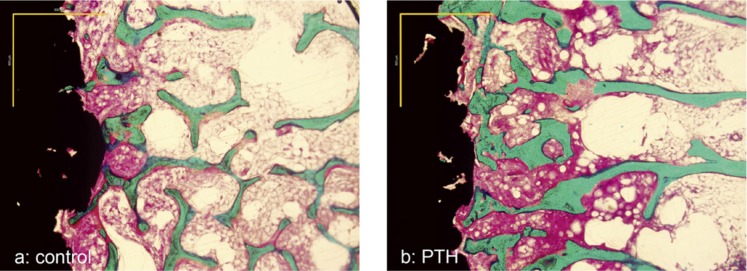
Photomicrographs of histological samples. Panel a: control. Panel b: parathyroid hormone (PTH). Scale: 900 μm. Staining technique: 0.4% basic fuchsin (red) and 2% light green (with bone staining green).

Woven bone was observed at the interface and also within the implant porosity in both treatment groups (Figure 4). In the PTH group, woven bone appeared to be thicker in trabecular structure. No implants had a detrimental dense fibrous membrane or cartilage.

### Histomorphometry

In the PTH group, woven bone in contact with the implant was 1.4 fold higher (significant) than in the control group (Table 1). At the implant surface, no difference was seen in lamellar bone or fibrous tissue. No differences in tissue volume of bone types and fibrous tissue were observed in the region immediately adjacent to the implant (0–500 μm) and in the concentric regions 500–1,000 μm and 1,000–2,000 μm of intact host bone outside the drill hole.

**Table:** Histomorphometry. Implant inserted press-fit in cancellous bone. Parathyroid hormone (PTH) treatment vs. control. Tissue fraction of bone, marrow–like tissue, and fibrous tissue at implant surface (surface fraction) and concentric region 0–500 μm, region 500–1,000 μm, and region 1,000–2,000 μm (volume fraction). n (PTH) = 8, n(control) = 9. Median (interquartile range)

	Woven bone	Lamellar bone	Marrow	Fibrous
Interface				
Control	11 (8–13)	6 (3–9)	84 (83–85)	0 (0–0)
PTH	15 (13–18)**^a^**	3 (2–5)	80 (76–82)**^a^**	0 (0–0)
Region 0–500 μm				
Control	10 (4–13)	13 (5–16)	78 (77–80)	0 (0–0)
PTH	14 (10–17)	8 (6–13)	75 (72–79)	0 (0–1)
Region 500–1,000 μm				
Control	4 (2–7)	20 (15–23)	74 (73–78)	0 (0–0)
PTH	7 (5–12)	13 (12–22)	77 (74–80)	0 (0–0)
Region 1,000–2,000 μm				
Control	2 (1–3)	18 (16–20)	80 (79–82)	0 (0–0)
PTH	3 (3–4)**^b^**	15 (11–16) **^b^**	83 (81–85)	0 (0–0)

**^a^** p < 0.05 (PTH compared to control). Mann-Whitney test.

**^b^** p = 0.07 within region (PTH compared to control). Mann-Whitney test.

### Mechanical testing

**Figure 5.**
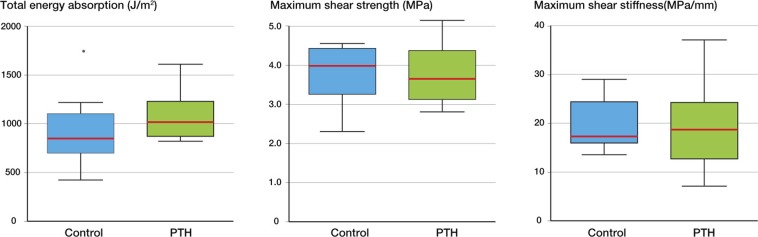
Results of mechanical testing; push-out to failure. Total energy absorption, maximum shear strength, and maximum shear stiffness. Box plots represent median, upper and lower quartiles, and sample range. n (PTH) = 8, n (control) = 8. * p < 0.05 (PTH compared to control). Mann-Whitney test.

We did not find any statistically significant differences between control implants and those treated with PTH regarding all mechanical parameters (Figure 5). Implants treated with PTH showed higher energy absorption with median (interquartile range) values of 1,017 (872–1,229) J/m^2^ for PTH and 850 (699–1,103) J/m^2^ for control. Median shear stiffness was 18.7 (12.7–24.2) MPa/mm in the PTH group and 17.3 (15.9–24.4) MPa/mm in the control group. In the PTH group, median shear strength was 3.7 (3.1–4.4) MPa and in the control group it was 4.0 (3.3–4.4) MPa.

## Discussion

The aim of this study was to establish the skeletal effect of systemic parathyroid hormone (PTH (1–34)) on cancellous bone growth and early mechanical fixation of unloaded porous coated implants inserted press-fit. Histomorphometric parameters revealed statistically improved woven bone at the implant surface. PTH did not increase bone volume in the 0–500 μm region and adjacent regions of intact bone, which had not been affected by the surgical preparation. Significantly higher mechanical parameters of shear stiffness, energy absorption, and shear strength were not achieved.

Implantation of uncemented orthopedic prostheses is performed press-fit in bone sites of (predominantly) trabecular bone. Subsequent osseointegration ensures the connection to the implant surface without intervening soft tissue. Cancellous osseointegration of an implant after administration of PTH (1–34) has not been studied previously in normal host bone. Limitations of our study included the experimental implant, the animal model, and the parathyroid hormone. Our experimental model allows evaluation of osseointegration of unloaded porous coated implants with the same surface treatment as found in human joint replacement (Soballe et al. 1990) and represents the proportion of human implants that are adjacent to cancellous bone. The PTH response was quantified by (1) unbiased stereological histomorphometry of undecalcified non-destroyed sections (implant in situ) and (2) mechanical testing of the combined static bone adherent to the implant surface and of the dynamic micro interlocking of bone adjacent to the porous surface during mechanical testing. The bone bed constituted intact non-pathological bone in juvenile dogs. The dog bone structure most closely resembles human bone (Aerssens et al. 1998) and is abundant in cancellous bone, enabling evaluation in this specific trabecular bone envelope with no cortical envelope contributing to the implant incorporation. The same sex of animals (male) reduces inter-individual biological variation in bone structure. The study design was unpaired due to systemic administration of PTH. Based on previous studies, a 4-week observation period is sufficient for evaluation of the early implant fixation in a press-fit implant model (Elmengaard et al. 2005). Our model was non-articular and the implant was not loaded during the gait cycle; thus, the effect of mechanical stimuli on bone formation alone or synergetic with PTH was not addressed (Sugiyama et al. 2008). We chose a single dose of PTH (1–34) (5 µg/kg body weight) and demonstrated efficacy of this dosage in increasing bone mass in cancellous bone with no statistically significant differences in lamellar bone. The dosage used is high in comparison to the clinical dose level of 20–40 µg per person per day evaluated in remodeling bone, but lower than in previous experimental implant studies in smaller animals (Skripitz and Aspenberg 2001a, b, Ohkawa et al. 2003, 2008. Shirota et al. 2003, Gabet et al. 2006, Dayer et al. 2007). The dosage is in accordance with other models in larger animal species and of relevance to dogs (Inoue et al. 1985, Boyce et al. 1996, Ma et al. 1997, Zhang et al. 1997, Jerome et al. 2001). However, we cannot rule out dose dependence in our results. 2 animals died unexpectedly. We were concerned about TFA, which is present in low concentration in research-grade PTH. Thus, as a precaution, from day 11 we changed the PTH to GMP-grade (same dosage, Bachem Certificate of Analysis). For each preparation, we used 5 µg/kg/day. All other animals completed the course uneventfully.

Intermittent administration of PTH (1–34) shows anabolic effects on bone by stimulating osteoblast number and bone formation (Mosekilde et al. 1991). In our dog model, intermittent PTH (1–34) administration of 5 µg/kg/day at 4 weeks improved the morphometric but not the mechanical parameters of implant fixation. Inserting implants press-fit confines the need for bone repair to the injured bone, which is the innermost bone along the implant surface. In the non-implanted surrounding bone, trabeculae are intact. The more extensive regenerating effect of PTH on bone is therefore restricted to the implant interface alone, with a quiescent response in the surrounding remodeling bone (Skripitz et al. 2000a, b). However, the bone formation at the implant surface caused a bone sealing along the implant with improved protection of the implant interface from subsequent loosening by the effect of joint fluid pressure and wear osteolysis. Although the mechanical parameters in the PTH group were higher, statistical significance was not achieved. Significance could be expected from longer observation time and implant loading (Sugiyama et al. 2008). The mechanical fixation of an implant relies on both the amount of peri-implant bone and architecture of bone trabeculae. Increased observation period may reveal increased fixation due to sufficient time to let tissue maturation occur at interface, for bone to remodel, and for improvement of the supporting non-injured peri-implant bone (Inoue et al. 1985, Nishida et al. 1994, Jerome et al. 1995, Zhang et al. 1997, Paschalis et al. 2003, 2005, Paschalis and Eriksen 2003). Long-term bone stimulation could also reduce the effect and consequence of stress shielding by preserving the host bone around the implant and thereby enhancing implant longevity.

PTH is a bone-building peptide with potential targets expanding far beyond the clinical application of osteoporosis (Seebach et al. 2004, Aleksyniene et al. 2006). Regenerating bone—as in fracture healing or in bone conduction chambers (Skripitz et al. 2000a, b)—appears to be more responsive to the anabolic actions of PTH than intact remodeling bone.

Research on the anabolic effect in joint replacements is experimental, and based on the rat model. In ovariectomized animals, rescue of bone loss before implantation is assumed with PTH (1–34) given at 30 μg/kg 3 times a week when administered for 20 weeks before and 2, 4, and 8 weeks after titanium screw insertion (Shirota et al. 2003). Commencing administration of PTH (1–34) at the time of implantation has revealed similar effects with 5, 25, and 75 μg/kg/day for 8 weeks (Gabet et al. 2006). When inserting titanium rods in tibial bone of protein-undernourished animals, PTH (1–34) at 40 μg/kg/day for 8 weeks improves osseointegration (Dayer et al. 2007). These studies were all conducted in rats models of pathological trabecular bone quality at the time of PTH administration—the initial low bone density inherent in these models gave room to bone improvement (Ohkawa et al. 2003, 2008). In normal bone, an increase in pull-out strength and removal torque has been described 4 weeks after implant insertion with daily injection of PTH (1–34) at 60 μg/kg (Skripitz and Aspenberg 2001b). With the same dose 3 times a week, pull-out strength was increased at 2 and 4 weeks, and implant-bone contact improved at 1, 2, and 4 weeks (Skripitz and Aspenberg 2001a). Implants for the mechanical testing were threaded stainless screws and histomorphometry was done destructively on stainless implant rods using decalcified sections, and defining bone-in-implant contact after removal of the implant. These and similar reports evaluated the effect on implants located transcortically with a substantial amount of cortical and subcortical bone contributing to the implant fixation (Skripitz and Aspenberg 2001a, b).

The anabolic response of PTH shows large variation between species (Jerome et al. 1995, Sietsema 1995, Whitfield et al. 1998, Tashjian and Chabner 2002), but also interskeletally between bone sites (Nishida et al. 1994, Jerome et al. 1995, 2001) and intraskeletally within the bone envelopes of cancellous, endocortical, cortical, and periosteal bone (Ejersted et al. 1994, Nishida et al. 1994, Jerome et al. 1995, Jilka et al. 2009). In models of smaller animals, PTH is a powerful bone anabolic agent. In larger animal species of clinical relevance and humans, the magnitude is less profound (Reeve 1996, Tashjian and Chabner 2002). In dog bone, we found significant osseointegration of press-fit orthopedic porous coated implants with the PTH (1–34) treatment. At this time point and dose of PTH (1-34), no significant increase in mechanical parameters was observed.

In conclusion, our findings support the concept that PTH (1–34) treatment improves histological cancellous osseointegration of orthopedic implants in normal bone. At the observed time point, no additional improvement of the initial mechanical fixation was observed. This was found in the context of porous coated titanium alloy implants inserted non-weight bearing and press-fit in cancellous bone of dogs. However, further studies on adjuvant PTH therapy are needed, concentrating on weight-bearing implants, PTH dosage, and longer treatment periods. Evaluation of long-term remodeling and growth of peri-implant bone stock is also warranted in the period after cessation of PTH treatment.
